# Comprehensive analysis of TGF-β-induced mRNAs and ncRNAs in hepatocellular carcinoma

**DOI:** 10.18632/aging.103826

**Published:** 2020-10-04

**Authors:** Junnan Liang, Jingyu Liao, Tongtong Liu, Yu Wang, Jingyuan Wen, Ning Cai, Zhao Huang, Weiqi Xu, Ganxun Li, Zeyang Ding, Bixiang Zhang

**Affiliations:** 1 Hepatic Surgery Center, Tongji Hospital, Tongji Medical College, Huazhong University of Science and Technology, Wuhan, China; 2 Department of Anesthesiology, Tongji Hospital, Tongji Medical College, Huazhong University of Science and Technology, Wuhan, China

**Keywords:** transforming growth factor β, epithelial-mesenchymal transition, hepatocellular carcinoma, competing endogenous RNAs

## Abstract

Transforming growth factor β (TGF-β) is a potent inducer of epithelial-mesenchymal transition (EMT) in hepatocellular carcinoma (HCC), and plays a critical role in its tumorigenesis and progression. Accumulating evidence indicates that protein-coding mRNAs, as well as non-coding RNAs (ncRNAs), may play key roles in the tumorigenesis and progression of HCC. In this study, we first report on the differential expression of lncRNAs, mRNAs, miRNAs, and circRNAs in Huh7 cells treated with TGF-β or DMSO for 7 days. Gene ontology (GO) and Kyoto Encyclopedia of Genes and Genomes (KEGG) pathway analyses were performed for significantly differentially expressed RNAs (DE RNAs). Then the competing endogenous RNA (ceRNA) network based on these DE RNAs was predicted and constructed. Among them, we identified that lncRNA SLC7A11-AS1 and hsa_circ_0006123 are involved in the EMT process induced by TGF-β and may promotes the metastasis of HCC. This knowledge may pave the way to develop novel clinical diagnostics and therapeutic approaches. Our study might open a new avenue for future investigations of the molecular mechanisms driving the EMT process induced by TGF-β in HCC.

## INTRODUCTION

Hepatocellular carcinoma (HCC) is one of the most common and aggressive human malignancies and the second leading cause of cancer-related deaths worldwide [1]. Effective treatments are limited due to the poor prognosis and high-recurrence rate, which is largely due to the high rate of tumor metastasis [2]. Therefore, improvements in HCC prevention and treatment strategies require a better understanding of the underlying pathophysiological mechanisms.

The transforming growth factors β(TGF-β) signaling pathway is a key player in tumorigenesis and cancer progression [3, 4]. TGF-β exerts a tumor-suppressive effect by inducing a cellular cytostatic program [5–7], but paradoxically, TGF-β is also known to function as a tumor promoter because of its prominent role in enhancing proliferation, migration and invasion [4, 8, 9]. It is well known that TGF-β promotes tumor invasion and metastasis in large part by inducing the epithelial-mesenchymal transition (EMT) [10, 11], which is a critical step in tumor invasion and dissemination [12, 13]. During the EMT process, cells lose some components of epithelial cell junctions and gain invasive properties [14]. TGF-β is a potent inducer of the EMT in HCC, and there are many underlying mechanisms that should be explored to fully understand the relationship between the TGF-β signaling pathway and EMT [15, 16].

Competing endogenous RNAs (ceRNAs) are RNAs that share miRNA recognition elements (MREs), and consequently compete for miRNA binding, contributing to mutual regulation lncRNAs, circRNAs, mRNAs, and pseudogenes [17, 18]. The lncRNAs are functionally defined as transcripts of more than 200 nucleotides in length with no protein coding potential [19]. The circRNAs are evolutionarily conserved transcripts featuring covalently linked 5‘ and 3’ ends that are derived from pre-mRNA back-splicing [20]. Numerous recent studies have demonstrated that ceRNAs play an important role in tumorigenesis and cancer progression by affecting the expression of key oncogenes or tumor suppressor genes. For example, a recent quantitative study demonstrated that ciRs7 acts as a sink that absorbs miR-7, influencing its target genes in many human cancers [21–23]. Jiang et al. reported that long noncoding RNA 00976 promotes pancreatic cancer progression through OTUD7B by sequencing miR-137 involved in the EGFR/MAPK pathway [24].

In this study, we performed high-throughput RNA-sequencing (RNA-Seq) in four paired Huh7 hepatoma cell cultures continuously treated with 10 ng/ml TGF-β or DMSO for 7 days to comprehensively identify differentially expressed lncRNAs, circRNAs, miRNAs, and mRNAs. Gene ontology (GO) and Kyoto Encyclopedia of Genes and Genomes (KEGG)pathway analyses were performed for the differentially expressed mRNAs (DE mRNAs) and target mRNAs of differentially expressed lncRNAs, miRNAs, and circRNAs. The ceRNA network in the EMT process of HCC induced by TGF-β will provide new insights into the potential therapeutics or novel diagnostics approaches for HCC. Then, we confirmed that the lncRNA SLC7A11-AS1 and hsa_circ_0006123 are induced by TGF-β and may be promotes the metastasis of HCC. Individual knockdown of lncRNA SLC7A11-AS1 or hsa_circ_0006123, significantly reduced the migration and invasion ability of HCC cells. Our study might offer a new avenue for future investigations to better understand the molecular mechanisms of the EMT process induced by TGF-β in HCC.

## RESULTS

### Identification of an EMT cell model

**Figure 1:**
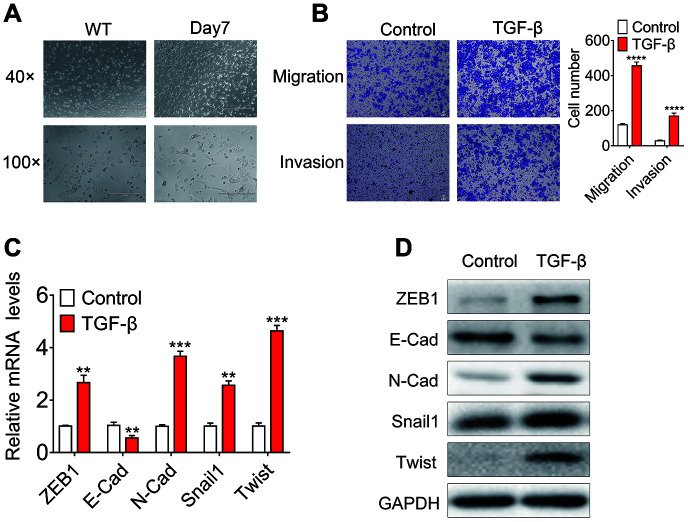
**Identification of the EMT cell model based on TGF-β treatment.** (**A**) Phase-contrast micrographs of Huh7 cells treated with 10 ng/ml TGF-β for 7 days or left untreated. Scale bar = 100 μm; (**B**) *In vitro* migration and invasion of Huh7 cells treated with 10 ng/ml TGF-β or left untreated. The number of migrated cells per field was counted after 48 h. (**C**) Relative mRNA levels of E-cadherin, ZEB1, SNAIL1, Twist and N-cadherin in Huh7 cells treated with 10 ng/ml TGF-β for 7 days or left untreated. (**D**) Relative protein levels of E-cadherin, ZEB1, SNAIL1, Twist and N-cadherin in Huh7 cells treated with 10 ng/ml TGF-β for 7 days or left untreated.

To identify ncRNAs that are regulated by TGF-β and involved in the EMT process induced by TGF-β, we treated huh7 and HLF cells with 10ng/ml TGF-β or DMSO for 7 days. TGF-β treated huh7 cells show a spindle-shipped appearance (Figure 1A). Furthermore, the *in vivo* migration and invasion ability of TGF-β treated cells was significantly increased (Figure 1B). Moreover, TGF-β treatments reduced the mRNA and protein expression levels of known epithelial-associated gene *E-cadherin*, while the expression of mesenchymal associated genes such as *ZEB1, SNAIL1, Twist* and *N-cadherin* was upregulated (Figure 1D, 1E). As shown in the (Supplementary Figure 1), Although TGF-β treated HLF cells did not show significant morphological differences, their *in vivo* migration and invasion ability were significantly increased. In the case of TGF-β stimulation, E-cadherin was downregulated and ZEB1, SNAIL1 and N-cadherin was upregulated.

### Identification of differentially expressed lncRNAs, mRNAs miRNAs and circRNAs in TGF-β-treated huh7 cells

**Figure 2:**
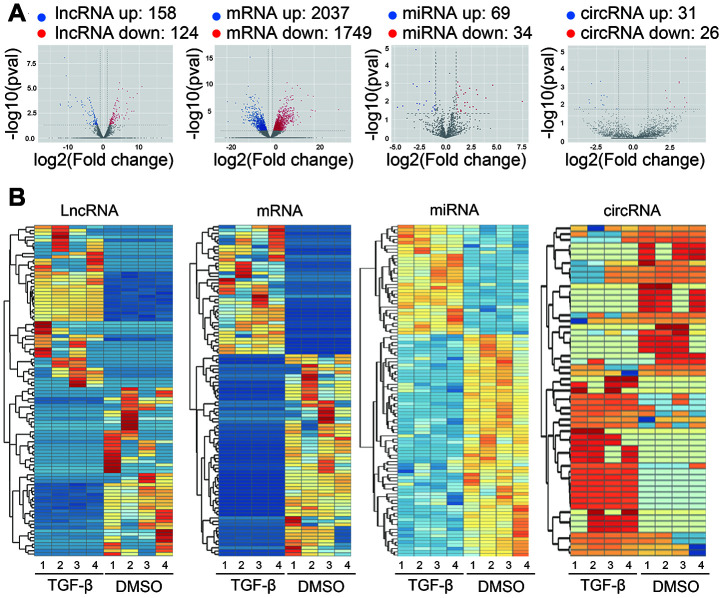
**Expression profiles of differentially expressed RNAs.** (**A**) Volcano plot showing differentially expressed lncRNAs, mRNAs, miRNAs and circRNAs with various p-values and fold changes. x axis: log2 ratio of RNA expression levels between treated and untreated Huh7 cells. y axis: false discovery rates (-log10 transformed) of different RNAs. Red points, upregulated RNAs; blue points, downregulated RNAs. (**B**) Hierarchical clustering analysis based on the significantly differentially expressed lncRNAs, mRNAs, miRNAs, and circRNAs, respectively (Fold Change > 2 and P-Value < 0.05). Red, high relative expression; blue, low relative expression; white, no change in gene expression. Color brightness reflects the degree of expression increase or decrease.

Second-generation sequencing technology was used to detected the differentially expressed lncRNAs, mRNAs, miRNAs and circRNAs between TGF-β-treated and DMSO treated Huh7 cells. Volcano plots were used to reveal differentially expressed lncRNAs, mRNAs miRNAs and circRNAs with various p-values and fold changes (Figure 2A). Among them, we identified 158 up- and 124 downregulated lncRNAs (Supplementary Table 1), 2037 up- and 1749 downregulated mRNAs (Supplementary Table 2), 69 up- and 34 downregulation miRNAs (Supplementary Table 3), as well as 31 up- and 26 downregulation circRNAs (Supplementary Table 4). Hierarchical clustering of these DE lncRNAs, DE mRNAs, DE miRNAs and DE circRNAs illustrated an obvious difference between TGF-β treated and DMSO treated huh7 cells (Figure 2B). Among the DE coding and non-coding RNAs, we uncovered 202 novel lncRNAs and 33 novel circRNAs that have not been reported before (Supplementary Tables 5, 6). These novel DEncRNAs suggest a promising new pattern in the EMT process induced by TGF-β in HCC.

### GO and KEGG enrichment analysis of differentially expressed lncRNAs, mRNAs, miRNAs and circRNAs

**Figure 3:**
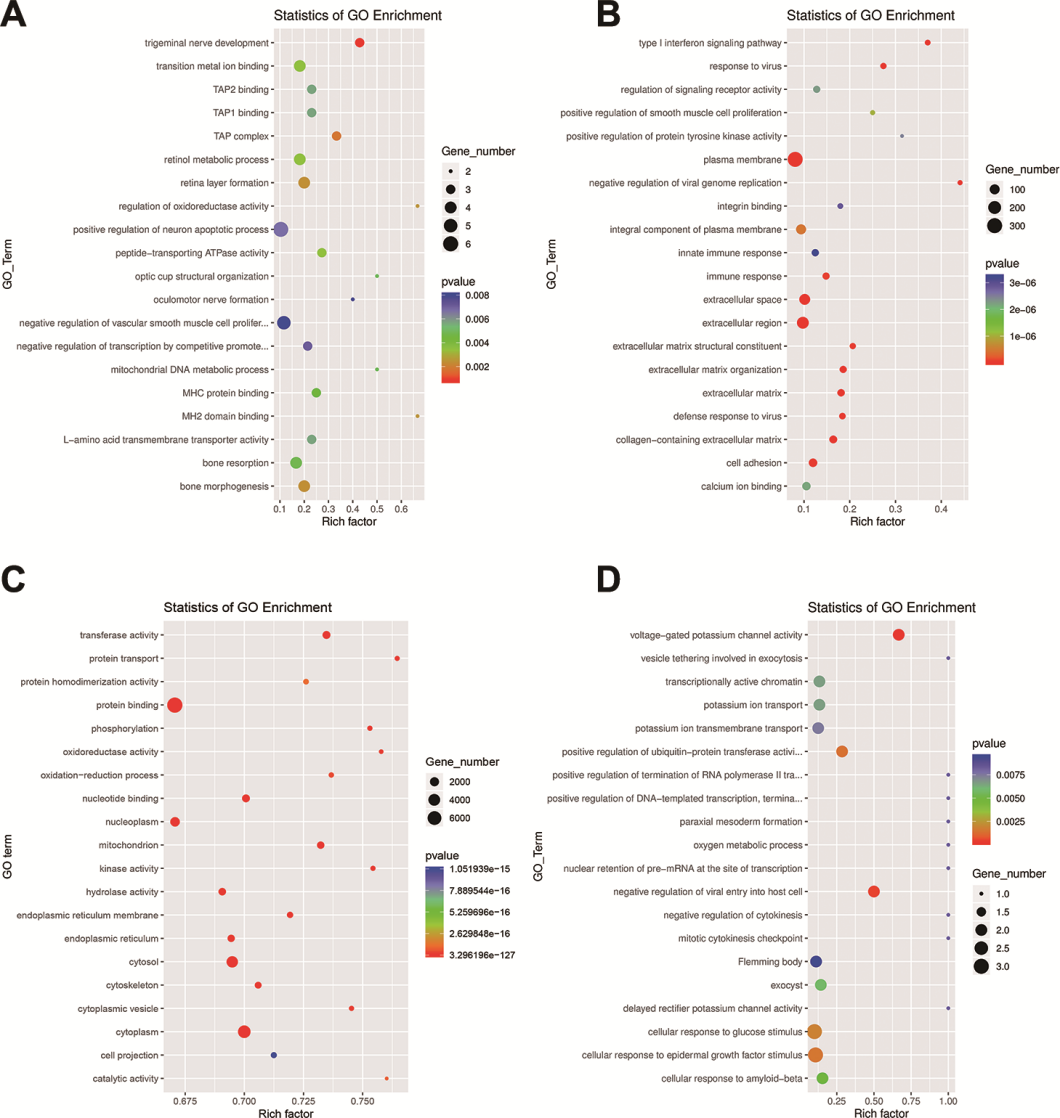
**GO analysis of dysregulated RNAs.** Top 20 GO terms for the differentially expressed lncRNAs (**A**), mRNAs (**B**), miRNAs (**C**) and circRNAs (**D**). The color and size of each circle represent the P-value and number of genes, respectively.

LncRNAs usually act on neighboring target genes, which is known as the cis role of lncRNAs [25, 26]. We searched for DE mRNAs derived from 100kb upstream and downstream of DE lncRNAs to predict putative cis target genes of DE lncRNAs, followed by analyzing the functions of these DE mRNAs to annotate the lncRNAs. The prediction results indicate that 282 lncRNAs may act on 450 target genes, forming a total of 754 lincRNA–gene connections (Supplementary Table 7). To explore the potential biological functions of these identified lncRNAs, we performed statistical enrichment of GO and KEGG terms among the target genes of the DE lncRNAs. Based on the results of GO analysis, several important GO terms were enriched, including “trigeminal nerve development”, “TAP complex”, “bone morphogenesis”, “retina layer formation”, and “regulation of oxidoreductase activity” (Figure 3A). KEGG pathway analysis showed that DE lncRNAs target genes are involved in several pathways such as, “neomycin”, “kanamycin” and “gentamicin biosynthesis”, “complement” and “coagulation cascades”, “riboflavin metabolism” and “staphylococcus aureus infection” (Supplementary Figure 2A). To predict the potential functional implications of the DE mRNAs, we performed GO and KEGG functional enrichment analyses. For DE mRNAs, the most relevant GO terms included “extracellular region”, “plasma membrane”, “type I interferon signaling pathway”, and” response to virus” (Figure 3B). The most relevant KEGG pathways were “PI3K-Akt signaling pathway”, “ECM-receptor interaction”, “small cell lung cancer” and “ovarian steroidogenesis” (Supplementary Figure 2B). To predict the potential functional implications of the DE miRNAs, we predicted target mRNAs of DE miRNAs using *Miranda* and *TargetScan*, after which GO and KEGG pathway enrichment analyses were conducted based on the target mRNAs. The result of GO enrichment analysis indicated that the DE miRNAs are closely associated with the terms “protein binding”, “cytoplasm”, “cytosol” and “transferase activity” (Figure 3C). Similarly, the data of KEGG pathway enrichment analysis indicated that the DE miRNAs were closely associated with the “p53 signaling pathway”, “pathways in cancer”, “pyrimidine metabolism” and “autophagy – animal” (Supplementary Figure 2C). Using the same approach, we also performed GO and KEGG analysis or the DE circRNAs, which suggested that the GO terms “voltage-gated potassium channel activity”, “negative regulation of viral entry into host cell”, and “positive regulation of ubiquitin-protein transferase activity” are enriched (Figure 3D), while “lysine degradation” was the only significantly enriched KEGG pathway (Supplementary Figure 2D). Many of the GO terms and KEGG pathway above have been reported correlated with EMT process, such as regulation of oxidoreductase activity, protein binding, transferase activity, positive regulation of ubiquitin-protein transferase activity, PI3K-Akt signaling pathway, p53 signaling pathway, pathways in cancer and so on [5, 27, 28].

### Validation of sequencing data using real-time PCR

**Figure 4:**
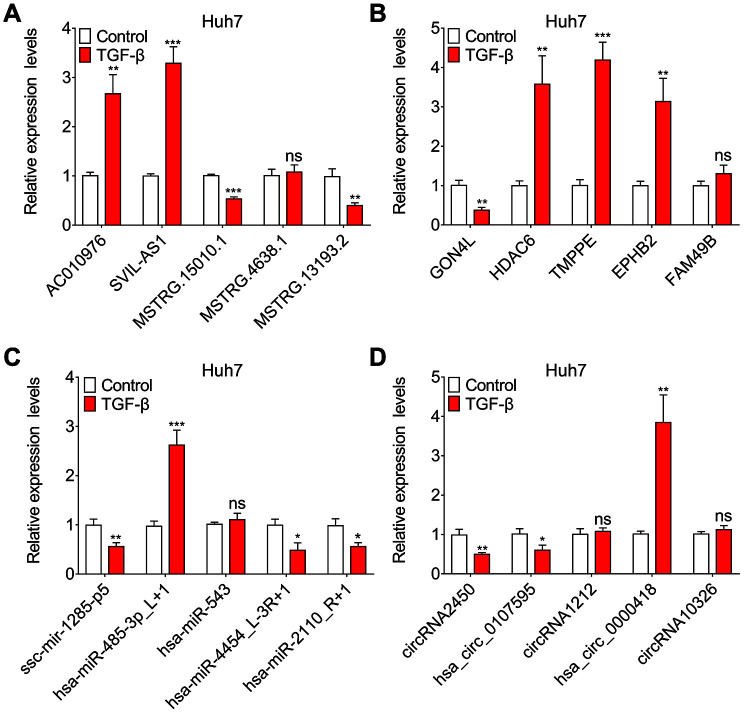
**Validation of sequencing data using qRT-PCR.** Relative expression levels of five randomly selected lncRNAs (**A**), mRNAs (**B**), miRNAs (**C**) and circRNAs (**D**), assessed by qRT-PCR.

We randomly selected differentially expressed transcripts for validation via qRT-PCR, including 5 DE lncRNAs, 5 DE mRNAs, 5 DE miRNAs and 5 DE circRNAs. The qRT-PCR was performed to determine the expression of these lncRNAs, mRNAs, miRNAs and circRNAs in paired TGF-β-treated and DMSO-treated Huh7 cell cultures. As shown in (Figure 4A–4D), the qRT-PCR results were consistent with the RNA-seq data.

### Identification of lncRNAs SLC7A11-AS1 and hsa_circ_0006123 as potentially related to the metastasis of HCC

**Figure 5:**
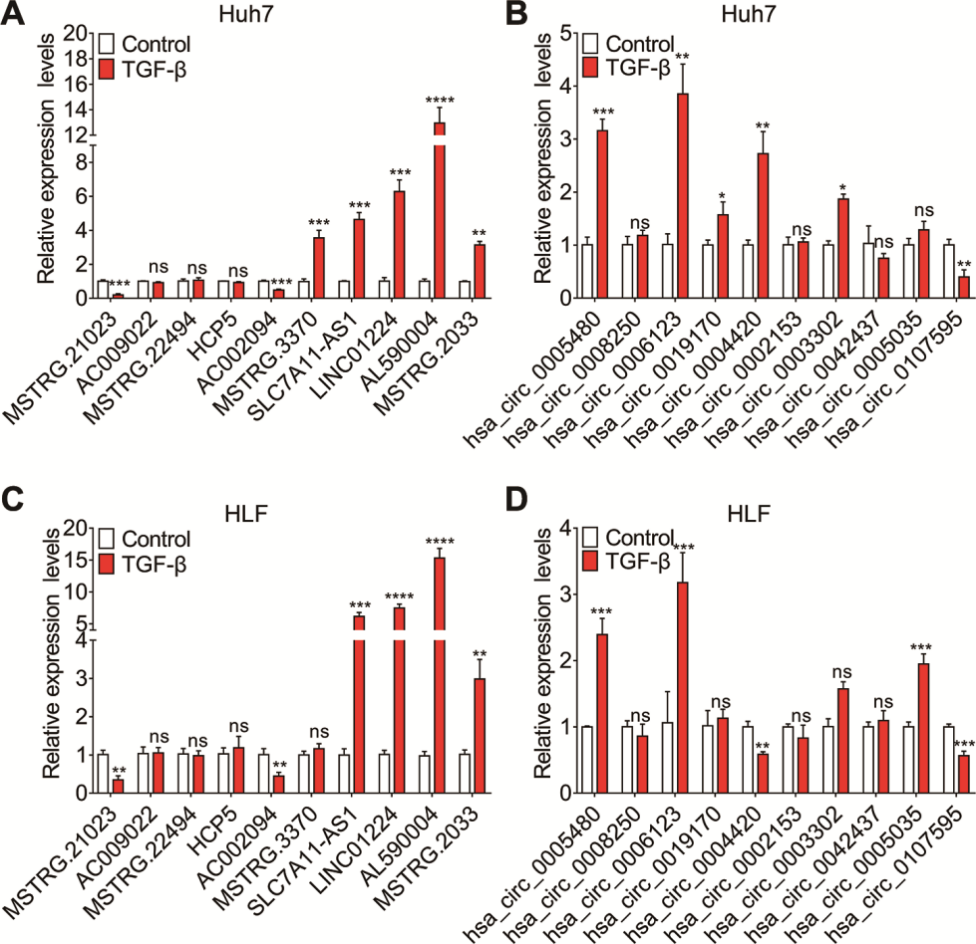
**Expression levels of the top ten dysregulated lncRNAs and known circRNAs assessed by quantitative real time-PCR (qRT-PCR).** Relative expression levels of ten dysregulated lncRNAs (**A**–**C**) and known circRNAs (**B**–**D**) in Huh7 and HLF cells treated with 10 ng/ml TGF-β for 7 days or left untreated.

**Figure 6:**
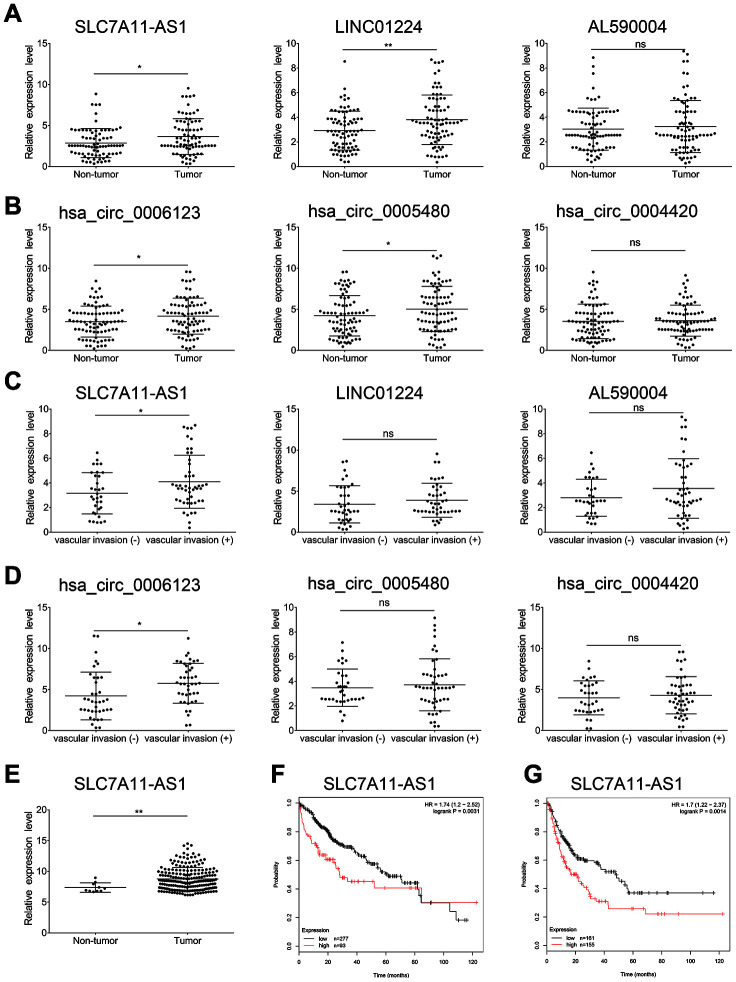
**The lncRNAs SLC7A11-AS1 and hsa_circ_0006123 may be related to the metastasis of HCC.** Expression levels of three selected lncRNA s (**A**) and three selected circRNAs (**B**) in 81 pairs of HCC samples and adjacent non-cancerous tissues using qRT-PCR. Expression levels of three selected lncRNAs (**C**) and three selected circRNAs (**D**) in patients with vascular invasion (Tumor-M) and patients without vascular invasion (Tumor-N). (**E**) Analysis of the lncRNA SLC7A11-AS1 expression level in TCGA liver cancer samples. (**F**) Kaplan–Meier analysis of overall survival in liver cancer patients stratified according to their SLC7A11-AS1 expression levels in the TCGA liver cancer cohort. (**G**) Kaplan–Meier analysis of diseases-free survival of liver cancer patients stratified according to their lncRNA SLC7A11-AS1 expression levels in the TCGA liver cancer cohort.

To screen lncRNAs and circRNAs that are involved in the TGF-β-induced EMT of HCC, we specifically selected the top 10 DE lncRNAs and known DE circRNAs for qRT-PCR validation in paired TGF-β-treated and DMSO-treated Huh7 and HLF cell cultures. The results indicated that 7 of 10 DE lncRNAs, as well as 6 of 10 known DE circRNAs were differentially expressed in the paired TGF-β-treated and DMSO-treated Huh7 cell cultures (Figure 5A and 5B). Similarly, 6 of 10 DE lncRNAs and 5 of 10 known DE circRNAs were differentially expressed in the paired TGF-β-treated and DMSO-treated HLF cell cultures (Figure 5C and 5D). Among these, we selected 3 DE lncRNAs and 3 DE circRNAs with the most significant expression differentials for further validation in 81 pairs of HCC samples and adjacent non-cancerous tissues using qRT-PCR. Among these six no-coding RNAs, lncRNA SLC7A11-AS1, LINC01224, hsa_circ_0006123 and hsa_circ_0005480 were found to be upregulated in HCC, while the expression of the others was similar to that in non-cancerous tissues (Figure 6A and 6B). Next, we divided the 81 patients into two groups based on the presence or absence of vascular invasion of the tumor. Reprocessing of the qRT-PCR data revealed that lncRNA SLC7A11-AS1 and hsa_circ_0006123 were upregulated in the patients with vascular invasion compared with the patients without vascular invasion. The other 4 no-coding RNAs did not show significant differences between the two groups of patients (Figure 6C and 6D). As shown in Table 1, overexpression of lncRNA SLC7A11-AS1 was related to vascular invasion and TNM stage. Moreover, overexpression of hsa_circ_0006123 was related to vascular invasion, TNM stage and BCLC stage. Baseline information on cancer history of all the cases are shown in Supplementary Table 11. Among them, the expression of the upregulated lncRNA SLC7A11-AS1 was confirmed by comparing with the TCGA database (Figure 6E). At the same time, Kaplan-Meier analysis using the TCGA database revealed that high lncRNA SLC7A11-AS1 levels in HCC tissues are significantly correlated with shorter overall survival (OS) (*P = 0.0031*; log-rank test; Figure 6F) and recurrence-free survival (RFS) *(P = 0.0014*; log-rank test; Figure 6G). Therefore, our data indicate that lncRNA SLC7A11-AS1 and hsa_circ_0005480 may play an important role in TGF-β-mediated HCC invasion and metastasis.

**Table 1:** Clinicopathologic characteristics of patients with hepatocellular carcinoma.

**Clinicopathological variables**	**SLC7A11-AS1 expression**		**P value**	**hsa_circ_0006123 expression**		**P value**
	Low (n=45)	High (n=36)		Low (n=38)	High (n=43)	
**Gender**						
Male	32	26	0.912	28	30	0.696
Female	13	10		10	13	
**Age**						
≤50	11	8	0.814	9	10	0.964
>50	34	28		29	33	
**AFP (ug/L)**						
≤20	13	10	0.762	11	12	0.916
>20	32	26		27	31	
**HBV**						
Negative	6	4	0.473	5	5	0.833
Positive	39	32		33	38	
**HCV**						
Negative	44	34	0.429	48	30	0.858
Positive	1	2		2	1	
**Tumor size (cm)**						
≤5	17	9	0.220	15	11	0.181
>5	28	27		23	32	
**Vascular invasion**						
No	23	10	**0.033**	21	12	**0.012**
Yes	22	26		17	31	
**Distant metastasis**						
No	45	34	0.109	38	41	0.178
Yes	0	2		0	2	
**Differentiation**						
I- II	28	27	0.220	24	31	0.353
III -IV	17	9		14	12	
**TNM stage**						
I	20	8	**0.036**	18	10	**0.022**
II -III -IV	25	28		20	33	
**BCLC stage**						
0+A	26	10	0.062	20	16	**0.030**
B+C	29	26		18	37	
**Adjuvant TACE**						
No	44	34	0.429	37	41	0.630
Yes	1	2		1	2	

### Construction and analysis of ceRNA networks of lncRNA LNCSLC7A11-AS1 and hsa_circ_0006123

**Figure 7:**
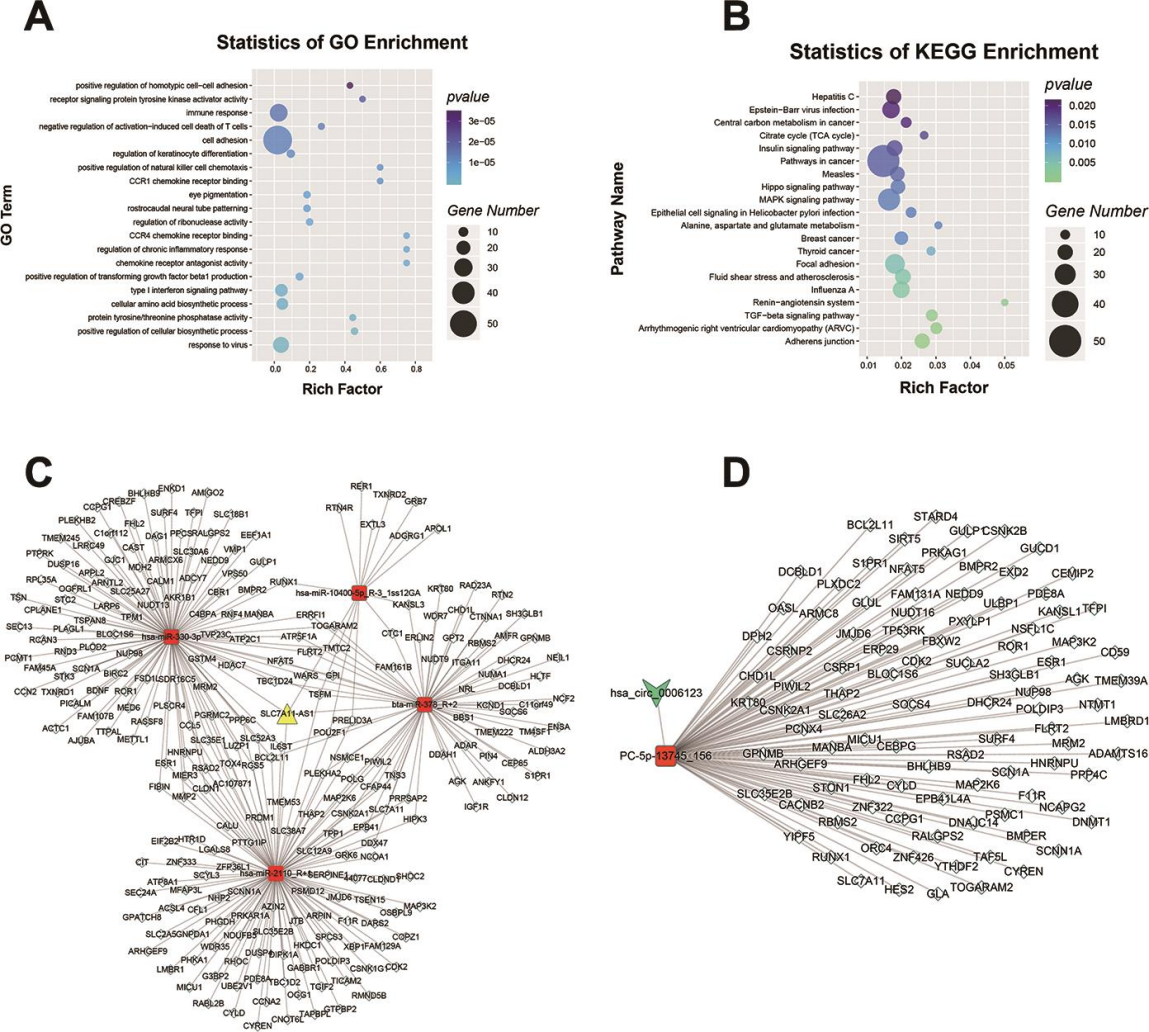
**LncRNA SLC7A11-AS1 and hsa_circ_0006123 ceRNA network.** GO (**A**) and KEGG (**B**) pathway analysis of the global ceRNA network. The ceRNA network of lncRNA SLC7A11-AS1 (**C**) and hsa_circ_0006123 (**D**) with largest degrees. The yellow nodes represent lncRNAs, the green nodes represent circRNAs, the red nodes represent miRNAs, and the white nodes represent mRNAs.

According to the ceRNA hypothesis, lncRNAs and circRNAs can sequester relevant miRNAs through MREs to post-transcriptionally regulate gene expression [18]. We used our RNA-seq data to construct ceRNA networks according to the interaction mechanism of lncRNA-miRNA-mRNA and circRNA-miRNA-mRNA complexes (Supplementary Table 8). GO analysis was performed, and several GO terms were found to be significantly enriched, including “type I interferon signaling pathway”, “cell cortex region” and “galactoside binding” (Figure 7A). KEGG pathway analysis showed that the terms “adherens junction”, “arrhythmogenic right ventricular cardiomyopathy” (ARVC) and “focal adhesion” were significantly enriched (Figure 7B). To further uncover the functions of lncRNA SLC7A11-AS1 and hsa_circ_0006123, two theoretical ceRNA networks derived from the global ceRNA network were predicted. The first network contained lncRNA SLC7A11-AS1, 4 downregulated miRNAs and 338 upregulated mRNAs (Figure 7C; Supplementary Table 9). The second network contained hsa_circ_0006123, 1 downregulated miRNA and 108 upregulated mRNAs (Figure 7D; Supplementary Table 10). GO (Supplementary Figure 3A and 3B), as well as KEGG pathway analyses (Supplementary Figure 3C and 3D) were performed to identify the potential functions of the two partial ceRNA networks. These results suggested that lncRNA SLC7A11-AS1 and hsa_circ_0006123 may function as ceRNAs that sequester miRNAs, thus implicating them in the pathogenesis of HCC.

### Knockdown of lncRNA SLC7A11-AS1 and hsa_circ_0006123 suppresses the migration and invasion of HCC cells

**Figure 8:**
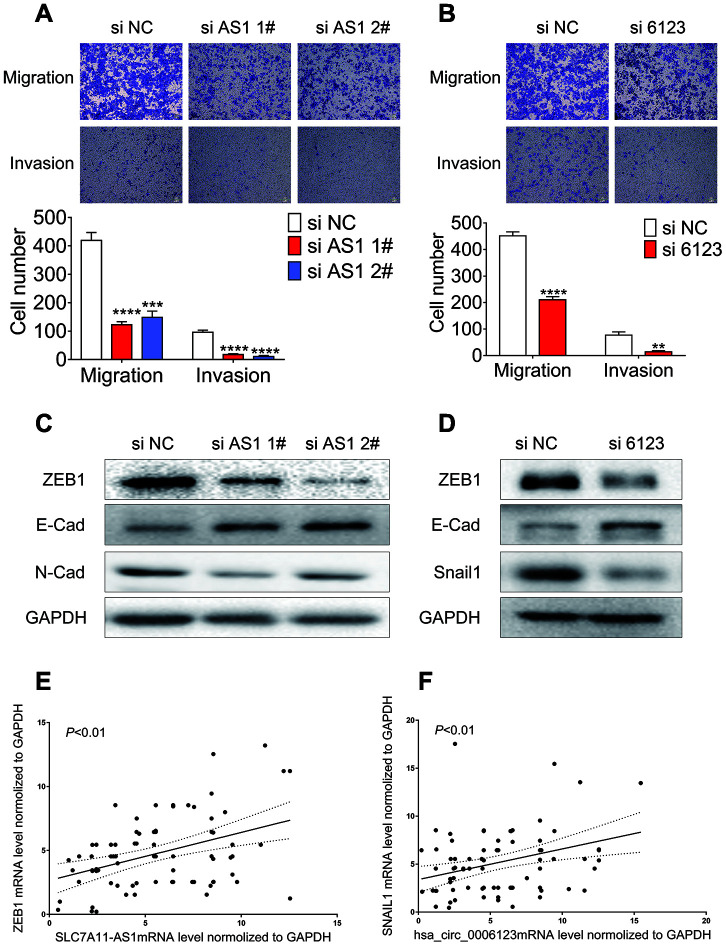
**Knockdown of lncRNA SLC7A11-AS1 and hsa_circ_0006123 suppresses the migration and invasion of Huh7 cell.** Migration and invasion of Huh7 following treatment with si-lncRNA SLC7A11-AS1 (**A**) or si-hsa_circ_0006123 (**B**). Relative protein levels of EMT markers in Huh7 cells following treatment with si-lncRNA LC7A11-AS1 (**C**) or si-hsa_circ_0006123 (**D**). qRT-PCR analysis showed that mRNA expression of lncRNA SLC7A11-AS1 and hsa_circ_0006123 were positively correlated with the ZEB1 (**E**) and snail1 (**F**) mRNA in 81 HCC patients

Based on the connection between higher levels of lncRNA SLC7A11-AS1 or hsa_circ_0006123 and vascular invasion in HCC patients, we further assessed the possible roles of lncRNA SLC7A11-AS1 and hsa_circ_0006123 in modulating HCC cell migration and invasion. Individual knockdown of lncRNA SLC7A11-AS1 or hsa_circ_0006123, significantly reduced the migration and invasion ability of Huh7 and HLF cells (Figure 8A, 8B and Supplementary Figure 4A, 4B). These results clearly demonstrate that the lncRNA SLC7A11-AS1 and hsa_circ_0006123 promote the migration and invasion ability of HCC cell lines. Moreover, knockdown of lncRNA SLC7A11-AS1 in Huh7 and HLF cells reduced protein expression levels of ZEB1 and N-cadherin, while the expression of E-cadherin was upregulated (Figure 8C and Supplementary Figure 4C). Knockdown of hsa_circ_0006123 increased E-cadherin while it decreased ZEB1 and snail1 in Huh7 and HLF cells. All these data indicated that lncRNA SLC7A11-AS1 and hsa_circ_0006123 promote EMT processes of HCC cells (Figure 8D and Supplementary Figure 4D). qRT-PCR analysis showed that mRNA expression of lncRNA SLC7A11-AS1 and hsa_circ_0006123 were positively correlated with the ZEB1 and snail1 mRNA in 81 HCC patients (Figure 8E, 8F).

## DISCUSSION

Although HCC is the third leading cause of cancer-related deaths worldwide [29], the underlying molecular mechanisms of its progression remain poorly characterized. The TGF-β signaling pathway has been increasingly recognized to be involved in tumorigenesis and progression of HCC [3]. The EMT is an essential event in cancer progression as well as a trigger of migration, invasion, and metastasis of HCC cells, whereby TGF-β is the strongest and most well-characterized stimulator of EMT and metastasis in HCC [4, 30]. TGF-β expression is usually elevated in many advanced or metastatic tumors, thus promoting the development of advanced tumors [31]. To explore the underlying molecular mechanisms of TGF-β-induced EMT in HCC, we constructed an EMT cell model by treating Huh7 cells with 10 ng/ml TGF-β or DMSO for 7 days. We artificially increase the concentration of TGF-β to simulate the environment in which TGF-β is highly expressed in advanced or metastatic tumors. Thus, the effect of TGFB on tumor cells can be further amplified to explore its mechanism of action. In addition to mRNAs, ncRNAs such as miRNAs, lncRNAs, and circRNAs were also demonstrated to play an essential role in the tumorigenesis and progression of HCC [32–34]. In fact, some pivotal TGF-β-regulated ncRNAs have been reported to be involved in tumorigenesis and cancer progression, including lncRNA-ATB, nc886, miR-29, miR-100 and miR-125b [15, 35–37]. However, these studies usually only focused on a single gene or protein in a single molecular pathway rather than on systematic or coordinated expression of other genes during HCC development. A comprehensive analysis of DE RNA profiles of HCC cells treated with TGF-β has, to our best knowledge, not been reported to date. To explore the functions and complex interactions of TGF-β-regulated ncRNAs and further explore and improve our knowledge of the TGF-β signaling pathway, we characterized the extensive transcription landscape related to the TGF-β-induced EMT process of HCC.

To screen lncRNAs and circRNAs that contribute to the TGF-β-induced EMT of HCC, we identified three most highly differentially expressed lncRNAs (lncRNA SLC7A11-AS1, LINC01224 and AL590004) and circRNAs (has_circ_0006123, has_circ_0005480 and has_circ_0004420). We found that lncRNA SLC7A11-AS1, LINC01224, hsa_circ_0006123 and hsa_circ_0005480 were upregulated in 81 pairs of HCC samples compared with adjacent non-cancerous tissues. The EMT induced by TGF-β is closely related to tumor metastasis, and further results showed that lncRNA SLC7A11-AS1 and hsa_circ_0006123 were upregulated in patients with vascular invasion compared with the patients without vascular invasion. LncRNA SLC7A11-AS1 has been reported to promote chemoresistance by blocking SCF^β-TRCP^-mediated degradation of *NRF2* in pancreatic cancer [38]. These results suggested that lncRNA SLC7A11-AS1 and hsa_circ_0006123 may be involved in the EMT process induced by TGF-β, and therefore closely associated with the metastasis of HCC.

The “ceRNA hypothesis”, which describes how mRNAs, transcribed pseudogenes, and lncRNAs “talk” to each other using MREs as letters of a new language, was first proposed by Salmena et al. [18]. Many lncRNAs and circRNAs have been reported to function as ceRNAs to sequester miRNAs and thereby further regulate the expression of mRNAs and their related proteins during carcinogenesis and tumor progression, including metastasis [15, 33]. Here, we showed for the first time that the ceRNA activity of these DE transcripts is related to the EMT induced by TGF-β in HCC cells (Supplementary Table 8). GO and KEGG pathway analyses were performed to identify the potential functions of the global ceRNA network. At the same time, a portion of the theoretical ceRNA network associated with lncRNA SLC7A11-AS1 and hsa_circ_0006123 was predicted. These results suggested that lncRNA SLC7A11-AS1 and hsa_circ_0006123 may act as ceRNAs.

Only a few ceRNAs were reported to be induced by TGF-β in HCC in earlier studies, and details of the ceRNA network induced by TGF-β in HCC remained unclear [15, 39, 40]. Here, we systematically investigated the ceRNA network based on differentially expressed lncRNAs, mRNAs, circRNAs and miRNAs for the first time. A part of these miRNAs had previously been linked to HCC, and they all target a number of lncRNAs, circRNAs and mRNAs, including the majority of the novel identified non-coding RNAs [41–44]. These findings illustrate the functional complexity of mRNAs and non-coding RNAs, and also indicate that these novel lncRNAs and circRNAs may be involved in the EMT process induced by TGF-β in HCC, meriting further investigation. Nevertheless, there are some limitations in our study. More complex animal experiment should be carried out to confirm that lncRNA SLC7A11-AS1 and hsa_circ_0006123 promote the migration and invasion ability of HCC cells in vivo. Furthermore, overexpression of the SLC7A11-AS1 and hsa_circ_0006123 lncRNAs should be performed to elucidate the underlying molecular mechanisms. It is possible that some of the differences in RNA expression found in this study are due to innate differences of cell lines. Our future work will focus on further investigating the molecular mechanisms of these ceRNAs, and exploring the role of the corresponding network in the progression and metastasis of HCC.

In conclusion, this is the first report on the differential expression of lncRNAs, mRNAs, miRNAs, and circRNAs in Huh7 cells treated with TGF-β or DMSO for 7 days. GO and KEGG pathway enrichment analyses, as well as constructing a ceRNA network allowed us to assess the roles and potential mechanisms of the differentially expressed transcripts. Among them, we identified that lncRNA SLC7A11-AS1 and hsa_circ_0006123 are likely involved in the EMT process induced by TGF-β, and may promotes the metastasis and of HCC. These findings will hopefully pave the way to develop novel clinical diagnostic and therapeutic approaches. Our study might therefore open a new avenue for future investigations of the molecular mechanisms driving the TGF-β-induced EMT of HCC.

## MATERIALS AND METHODS

### Patients and specimens

This study included patients with human primary liver cancer who underwent liver resection at the Hepatic Surgery Center of Tongji Hospital between Sept 2012 and Oct 2017 and who provided their informed consent. The study was approved by the Medical Ethics Commission of Medical Ethics Committee of Tongji Hospital.

### Cell lines and culture

Huh7 and HLF cell lines were purchased from the China Center for Type Culture Collection (Wuhan, China). They are cultured in Dulbecco's modified Eagle's medium (Invitrogen Corporation, Carlsbad, CA, USA) supplemented with 10% FBS (Life Technologies Inc., Gibco/Brl Division, Grand Island, NY, USA) under the culture conditions of 37°C and 5% CO2.

### Construction of EMT cell model

According to the previous experimental results and recent detection results in our laboratory, we found that the TGF-β expression levels of Huh7 and HLF were relatively low. In addition, Huh7 show a well-differentiated epithelial morphology [45]. Therefore, we believe that they have greater potential for TGF-β induced EMT. To establish the model of cancer cells undergoing EMT, cells were treated with 10 ng/ml TGF-β for 7 days with TGF-β replenishment every day [46]. Compared with HLF, Huh7 showed more obvious morphological and migration changes after TGF-β treatment. Therefore, we chose Huh7 for the sequencing.

### RNA library construction and sequencing

Total RNA was isolated and purified using Trizol reagent (Invitrogen, Carlsbad, CA, USA) following the manufacturer's procedure. The RNA amount and purity of each sample was quantified using NanoDrop ND-1000 (NanoDrop, Wilmington, DE, USA). The RNA integrity was assessed by Agilent 2100 with RIN number >7.0. Approximately 5 ug of total RNA was used to deplete ribosomal RNA according to the manuscript of the Ribo-Zero™ rRNA Removal Kit (Illumina, San Diego, USA). After removing ribosomal RNAs, the left RNAs were fragmented into small pieces using divalent cations under high temperature. Then the cleaved RNA fragments were reverse-transcribed to create the cDNA, which were next used to synthesize U-labeled second-stranded DNAs with E. coli DNA polymerase I, RNase H and dUTP.. An A-base is then added to the blunt ends of each strand, preparing them for ligation to the indexed adapters. Each adapter contains a T-base overhang for ligating the adapter to the A-tailed fragmented DNA. Single-or dual-index adapters are ligated to the fragments, and size selection was performed with AMPureXP beads. After the heat-labile UDG enzyme treatment of the U-labeled second-stranded DNAs, The ligated products are amplified with PCR by the following conditions: initial denaturation at 95°C for 3 min; 8cycles of denaturation at 98°C for 15 sec, annealing at 60°C for 15 sec, and extension at 72°C for 30 sec; and then final extension at 72°C for 5 min. The average insert size for the final cDNA library was 300 bp (±50 bp). At last, we performed the paired-end sequencing on an IlluminaX Ten (LC Bio, China) following the vendor's recommended protocol.

### Different expression analysis of lncRNAs, mRNAs, miRNAs and cicrRNAs

StringTie was used to perform expression level for lncRNAs, mRNA and miRNA by calculating FPKM. The differentially expressed mRNAs and lncRNAs were selected with log2 (fold change) >1 or log2 (fold change) <-1 and with statistical significance (p value < 0.05) by R package Ballgown [47].

### Quantitative real-time (qRT)-PCR

Trizol regent (Invitrogen) was used to extracted total RNA, and FastQuant cDNA Synthesis Kit (TIANGEN, Beijing, China) was used to performed reverse transcription from 2 μg total RNAs. Quantitative real-time PCR was carried out with SuperReal PreMix Plus (SYBR Green, TIANGEN, Beijing, China) according to the manufacture’s instruction on a CFX Connect™ Real-Time PCR Detection System (Bio-Rad, Hercules, CA, USA). For all qRT-PCR experiments, data analysis was performed byΔΔCt method and GAPDH was used as controls.

### Migration and invasion assays

For transwell assays, cells were trypsinized and resuspended in culture medium without FBS after transfected with 100 nM gene-speicific siRNA (RiboBio, China) or non-targeting control employing Lipo3000 (Invitrogen) following the manufacturer’s instruction. For migration assay, 5–10 × 104 normal liver cells or HCC cells were added to the top 8-μm chamber without Matrigel. For invasion assays, 10–20 × 104 cells were added to the Matrigel-coated upper chamber. The lower chambers were filled with medium containing 10–20% serum. After 24–48 h incubation, the invaded cells at the bottom surface of the upper chamber membrane were fixed in 4% paraformaldehyde, stained with 1% crystal violet and counted under a microscope in a blinded manner.

### Gene ontology and kyoto encyclopedia of genes and genomes analysis

Gene Ontology (GO) analysis annotates differentially expressed genes in terms of cell composition, molecular function, and biological processes. Kyoto Encyclopedia of Genes and Genomes (KEGG) analysis is an effective method for predicting the potential biological function of differentially expressed genes. Gene Ontology (GO) classification and the Kyoto Encyclopedia of Genes and Genomes (KEGG) pathway enrichment were conducted using the clusterProfiler of R/Bioconductor1. In the GO and KEGG analyses, P value < 0.05 was used as the screening standard.

### ceRNA network analysis

We searched the sequences of the lncRNAs, circRNAs and mRNAs to identify potential MREs. We used miRanda (http://www.microrna.org/microrna/home.do) to predict miRNA-binding seed-sequence sites, and an overlap of the same miRNA-binding sites on both circRNAs and mRNAs indicated potential circRNAmiRNA-mRNA interaction. ceRNA networks were constructed and visually displayed using Cytoscape software v.3.5.0 (San Diego, CA, USA).

### Statistical analysis

All statistical analyses were performed using GraphPad Prism 7.0 and Statistical Program for Social Science version 22 (SPSS 22.0). All the statistical data on normal distribution are displayed as mean value ± standard deviation and and were compared with t-test. Data on abnormal distribution were compared with a nonparametric test. Clinical correlations were analyzed using the v2 test. All statistical tests were 2-sided, and p < 0.05 was regarded as statistical significance.

## Supplementary Material

Supplementary Figures

Supplementary Table 1

Supplementary Table 2

Supplementary Table 3

Supplementary Table 4

Supplementary Table 5

Supplementary Table 6

Supplementary Table 7

Supplementary Table 8

Supplementary Table 9

Supplementary Table 10

Supplementary Table 11
