# Supplementary material for: Comprehensive analysis of TGF-β-induced mRNAs and ncRNAs in hepatocellular carcinoma
Source: Aging (Albany NY). 2020 Oct 4;12(19):19399–420. doi: 10.18632/aging.103826 (PMC7732333; doi:10.18632/aging.103826)
Supplement: Supplementary Figures [file aging-12-103826-s001..pdf]

SUPPLEMENTARY FIGURES

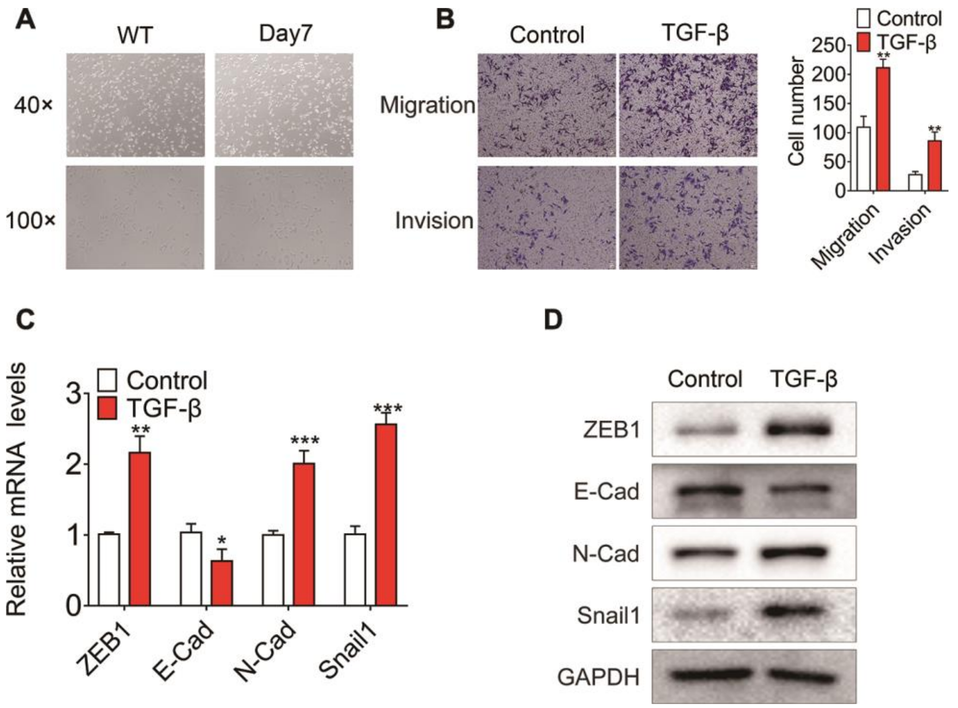

Supplementary Figure 1. Identification of the EMT cell model based on TGF-β treatment. (**A**) Phase-contrast micrographs of HLF cells treated with 10 ng/ml TGF-β for 7 days or left untreated. Scale bar = 100 µm; (**B**) In vitro migration and invasion of HLF cells treated with 10 ng/ml TGF-β or left untreated. The number of migrated cells per field was counted after 48 h. (**C**) Relative mRNA levels of E-cadherin, ZEB1, SNAIL1, and N-cadherin in HLF cells treated with 10 ng/ml TGF-β for 7 days or left untreated. (**D**) Relative protein levels of E-cadherin, ZEB1, SNAIL1 and N-cadherin in HLF cells treated with 10 ng/ml TGF-β for 7 days or left untreated.

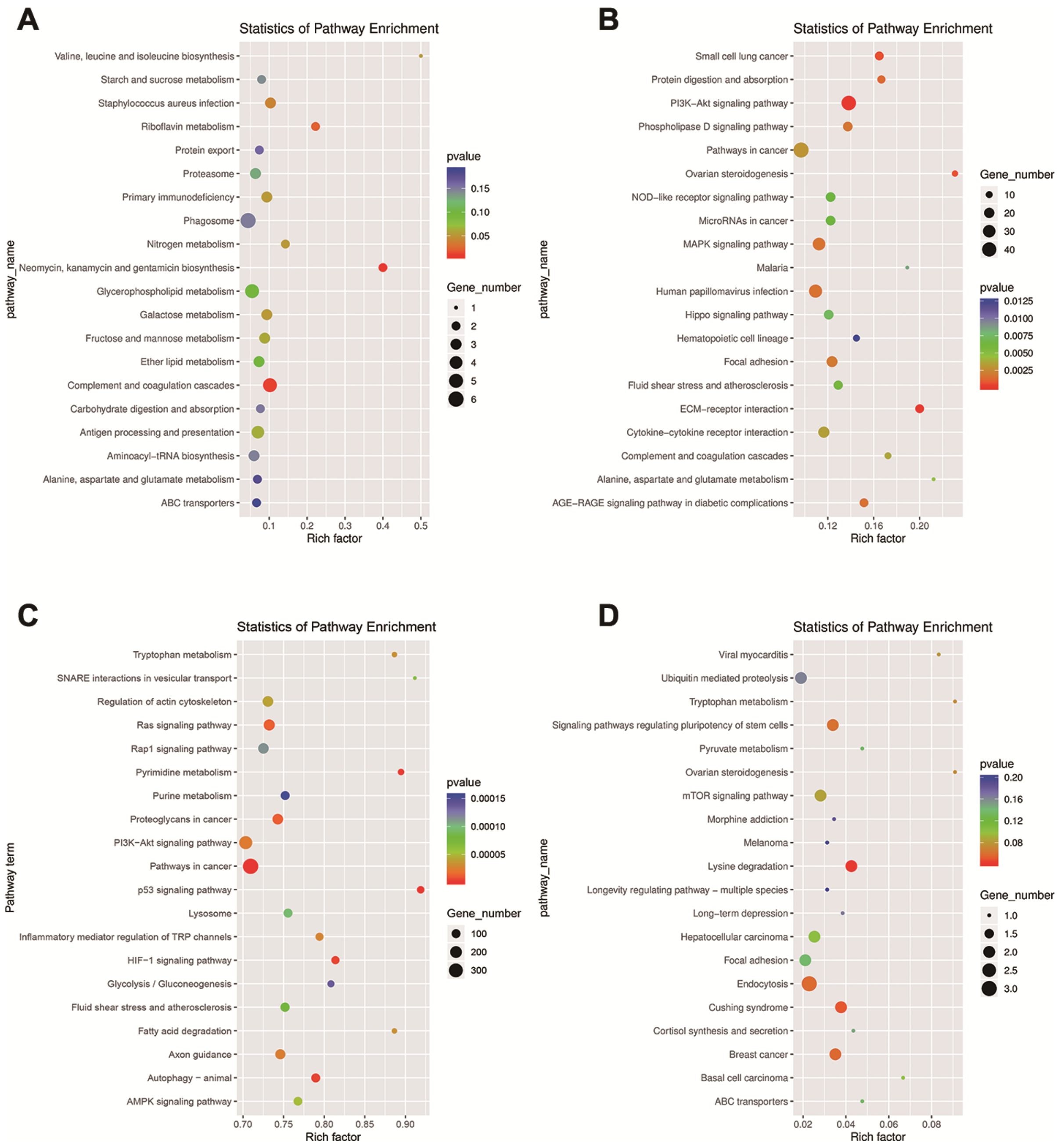

Supplementary Figure 2. KEGG pathway analysis of dysregulated RNAs. Top 20 KEGG pathways for the differentially expressed lncRNAs (**A**), mRNAs (**B**), miRNAs (**C**) and circRNAs (**D**). The color and size of each circle represents the P-value and the number of genes, respectively.

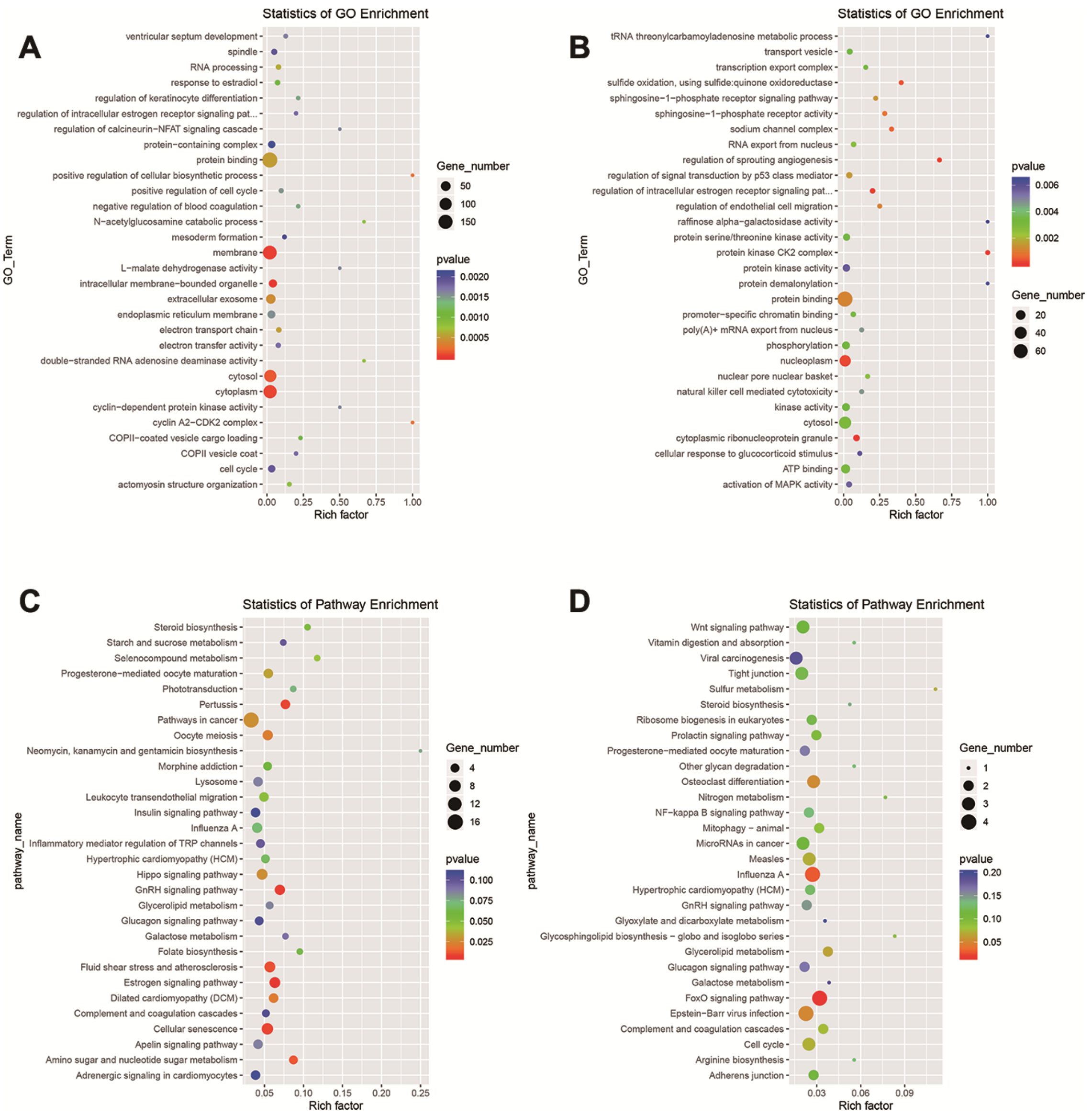

Supplementary Figure 3. GO and KEGG pathway analysis of two partial ceRNA networks. GO enrichment analysis for the modules of the lncRNA SLC7A11-AS1 (**A**) and hsa_circ_0006123 (**B**) ceRNA networks. KEGG pathway enrichment analysis for the modules of the lncRNA SLC7A11-AS1 (**C**) and hsa_circ_0006123 (**D**) ceRNA networks.

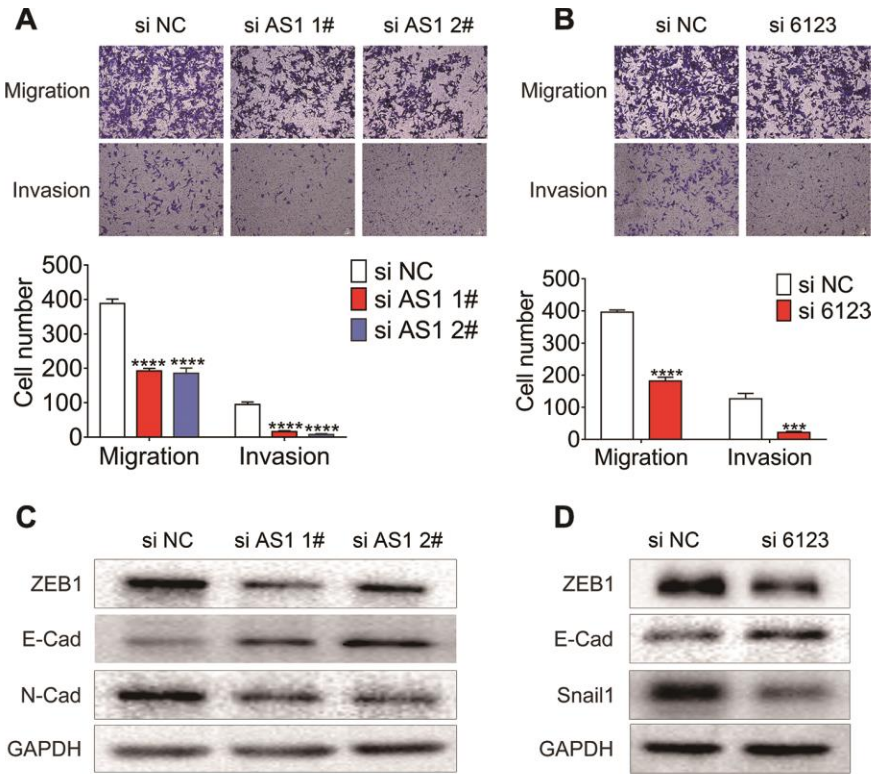

Supplementary Figure 4. Knockdown of lncRNA SLC7A11-AS1 and hsa_circ_0006123 suppresses the migration and invasion of HLF cell. Migration and invasion of HLF following treatment with si-lncRNA SLC7A11-AS1 (**A**) or si-hsa_circ_0006123 (**B**). Relative protein levels of EMT markers in HLF cells following treatment with si-lncRNA LC7A11-AS1 (**C**) or si-hsa_circ_0006123 (**D**).
