# Supplementary material for: Comprehensive analysis of TGF-β-induced mRNAs and ncRNAs in hepatocellular carcinoma
Source: Aging (Albany NY). 2020 Oct 4;12(19):19399–420. doi: 10.18632/aging.103826 (PMC7732333; doi:10.18632/aging.103826)
Supplement: Supplementary Table 11 [file aging-12-103826-s012..pdf]

SUPPLEMENTARY TABLE

Supplementary Table 11. Clinicopathologic characteristics of patients with hepatocellular carcinoma.

Clinicopathological variables	Number N=81	Percentage
**Gender**		
Male	58	71.6
Female	23	28.4
**Age**		
≤50	19	23.5
>50	62	76.5
**AFP (ug/L)**		
≤20	23	28.4
>20	58	71.6
**HBV**		
Negative	10	12.3
Positive	71	87.7
**HCV**		
Negative	78	96.3
Positive	3	3.7
**Tumor size (cm)**		
≤5	26	32.1
>5	55	67.9
**Vascular invasion**		
No	33	40.7
Yes	48	59.3
**Distant metastasis**		
No	79	97.5
Yes	2	2.5
**Differentiation**		
I- II	55	67.9
III -IV	26	32.1
**TNM stage**		
I	28	34.5
II -III -IV	53	65.5
**BCLC stage**		
0+A	36	44.4
B+C	45	55.6
**Adjuvant TACE**		
No	78	96.3
Yes	3	3.7
